# Serial Cell-free DNA Assessments in Preclinical Models

**DOI:** 10.1016/j.xpro.2020.100145

**Published:** 2020-10-20

**Authors:** Ariana Rostami, Caberry Yu, Scott V. Bratman

**Affiliations:** 1 Princess Margaret Cancer Center, University Health Network, 101 College Street, Toronto, ON M5G 1L7, Canada; 2 Department of Medical Biophysics, University of Toronto, 101 College Street, Toronto, ON M5G 1L7, Canada; 3 Department of Radiation Oncology, University of Toronto, 149 College Street, Toronto, ON M5T 1P5, Canada

## Abstract

Studying circulating cell-free DNA (cfDNA) release within preclinical model systems provides opportunities to investigate the mechanisms and kinetics underlying this process under various conditions. We present a detailed protocol for longitudinal evaluation of cfDNA release through (1) seeding of cancer cell lines and establishment of xenograft tumors, (2) treatment of cancer cells and xenograft tumors, (3) serial collection of cell line media and xenograft blood, and (4) processing and isolation of cfDNA for (5) quantification of cfDNA by quantitative PCR.

For complete details on the use and execution of this protocol please refer to Rostami et al. (2020).

## Highlights

• Cell line media and xenograft blood can be serially collected after irradiation
• Small plasma volumes can be simultaneously purified for cell-free DNA (cfDNA)
• Cell line media can be diluted for quantification of cfDNA using quantitative PCR
• Human LINE-1 can be used for ultrasensitive detection of cfDNA

## Graphical Abstract

**Figure:**
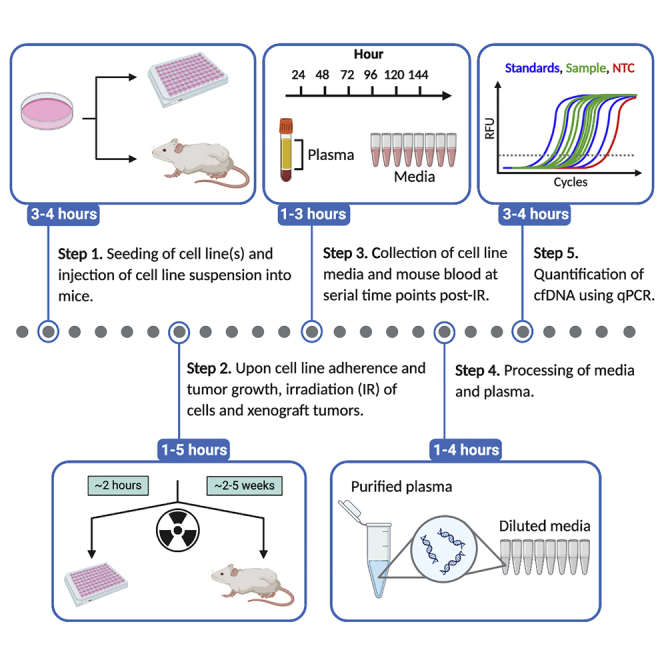

## Before You Begin

### Injection of Cell Line Suspensions into NSG Mice and Blood Collection

Below we describe the protocol for using Cal33 cells but have also successfully used the protocol with other head and neck squamous cell carcinoma (HNSCC) cell types (i.e., HMS-001, Vu147T, FaDu) and lung adenocarcinoma cell types (i.e., PC-9 and HCC-827).

***Note:*** All mouse experiments and blood collection schedules must be performed with the approval of an Animal Care Committee that is relevant to your institution.

***Note:*** Use immunocompromised mice for injection of cell lines. We recommend using NOD-SCID-IL2Rγ^null^ (NSG) mice (6–8 weeks old) since they have a high success rate for tumor engraftment. Supplier of NSG mice is the Jackson Laboratory (https://www.jax.org).

***Alternatives:*** NOD-SCID or NOD-Rag1^null^-IL2rγ^null^ (NRG) can be used, depending on the cell line and intended treatment condition. The use of immunocompromised mice limits the ability to assess the function of the immune system in modulating treatment-induced cfDNA release and degradation. As discussed in Rostami et al. (2020), our models focused on the early release of ctDNA and cell death, before immune-mediated cell death is likely to take place (Schneider et al. 2017). However, the degradation and clearance of cfDNA by immune cells in circulation cannot be evaluated in this model. This limitation should be considered when utilizing these models for cfDNA analysis.

***Note:*** An isoflurane chamber with nose cone attachment is required to anesthetize the mice.

### Diluting Media

***Note:*** This protocol describes a method for diluting media into DNA suspension buffer for efficient downstream quantitative PCR (qPCR) analysis. Media is diluted with a minimum dilution of 1/100 or higher in order to avoid qPCR inhibition from media components. This allows for qPCR-based quantification of cfDNA without the need for DNA precipitation or column-based purification.

***Note:*** This protocol has only been tested with the following media (DMEM, RPMI, DMEM/F12, IMDM), and caution should be taken when using the protocol with other media types.

***Note:*** This protocol utilizes SYBR Green Supermix with Sso7d fusion DNA polymerase. Caution should be taken when using other DNA polymerases, as compatibility cannot be guaranteed.

## Key Resources Table

**Table:** 

REAGENT or RESOURCE	SOURCE	IDENTIFIER
**Chemicals, Peptides, and Recombinant Proteins**		

Phosphate Buffered Saline (PBS)	Wisent	311-010-CL
0.25% Trypsin	Wisent	325-043-EL
Trypan Blue	Gibco	15250-061
Penicillin/Streptomycin (10,000 U/mL)	Wisent	450-200-EL
Fetal Bovine Serum	Gibco	A31607-01
Dulbecco’s Modified Eagle Media (DMEM)	Wisent	319-005-CL
DNA Suspension Buffer	TEKnova	T0220
EB Buffer	Qiagen	19086
Matrigel Matrix Basement Matrix	Corning	356231
Proteinase K	Qiagen	Cat#1018332
Buffer ACL	Qiagen	Cat#939017
Buffer ACB	Qiagen	Cat#1069275
Saline	Baxter	JB1423

**Critical Commercial Assays**		

CaspaseGlo®3/7	Promega	G8090
Presto Blue	Thermo Fisher	A13262
QIAmp 96 DNA Blood Kit	Qiagen	Cat#51161
Sso Advanced Universal SYBR Green Supermix	Bio-Rad Laboratories	172-5274
Human Genomic DNA	Promega	G147A

**Experimental Models: Organisms/Strains**		

Mouse: Nod-Scid-Gamma	The Jackson Laboratory	Cat#005557

**Experimental Models: Cell Lines**		

Cal33	DSMZ	ACC-447, CVCL_1108

**Oligonucleotides**		

Short human LINE-1 Forward Primer: TCACTCAAAGCCGCTCAACTAC	Eurofins Genomics	N/A
Short human LINE-1 Reverse Primer: TCTGCCTTCATTTCGTTATGTACC	Eurofins Genomics	N/A
Long human LINE-1 Forward Primer: TCTGCCTTCATTTCGTTATGTACC	Eurofins Genomics	N/A
Long human LINE-1 Reverse Primer: TCAGCACCACACCACACCTATTC	Eurofins Genomics	N/A

**Other**		

IGRT-XRAD 225Cs Irradiator	Precision X-ray	N/A
Gammacell® 40 Exactor (^137^Cs irradiator)	Best Theratronics	N/A
Hemacytometer	VWR	15170-170
Hemacytometer cover glass (26 × 20 mm) (1 × 7/8 inch)	VWR	16007-111
Evos-XL microscope	Invitrogen	AME3300
Microplate shaker	VWR	Cat#12620-926
TECAN Infinite M200Pro Plate Reader	TECAN	N/A
8-channel Multichannel Pipette (30–300 μL)	Eppendorf	Cat#3125000052
8-channel Multichannel Pipette (0.5–10 μL)	Eppendorf	Cat#3125000010
15 mL Falcon Tube	ESBE Scientific	LBC-31313450090
50 mL Falcon Tube	ESBE Scientific	LBC-31813450090
1.5 mL Snap Cap Microtubes	DiaMed	PRE150-N
8-strip PCR tubes	DiaMed	diatec420-1001
Viaflo Electronic Pipette	Integra	Part#4011
Griptips	Integra	Part#4425
Repeater® M4	Eppendorf	Cat#4982000322
Combitips advanced® 0.1 mL	Eppendorf	Cat#0030089618
Microseal® B PCR Plate Sealing Film	Bio-Rad	Cat#msb1001
FrameStar 384 Well Skirted PCR Plate	4titude	4ti-0387
CFX384 Touch Real-Time PCR Detection system	Bio-Rad	Cat#698-2901
NucleoVac 96 Vacuum Manifold	Takara	Cat#740681
Isoflurane chamber with nose cone	Benson Medical	T3ISO
Ear Notcher	Fine Science Tools	24210-02
0.5 mL syringe (permanently attached needle)	BD Science	305620
1 mL syringe	BD Science	309659
PrecisionGlide Needle 25G	BD Science	305122
Microcuvette CB 300 tubes coated with EDTA	Sarstedt	16.444.100
Goldenrod 5 mm lancet	VWR	MSPP-GR5MM

## Materials and Equipment

***Alternatives:*** This protocol describes the qPCR procedure using a CFX384 Touch Real-Time PCR Detection system and corresponding reagents, in 10 μL reaction volumes. Alternative qPCR systems can be used as well. We recommend using a 384-well format, since this allows examination of multiple samples across timepoints in the same plate, thus minimizing potential batch effects.

## Step-By-Step Method Details

### Seeding Cell Lines and Treatment

**Timing: 3–4 h**

This section describes the steps used to seed human cancer cell lines into 96-well plates prior to treatment with irradiation (or other anti-cancer agents of choice).

***Note:*** If only interested in measuring cell viability, use a black 96-well clear bottom plate. If interested in measuring caspase activity, use a white 96-well clear bottom plate. Assessment of cell viability, caspase activity, and media collection will occur in the same 96-well plate.

***Note:*** Use an appropriate number of replicates. We recommend using at least three biological replicates, if possible. Ensure there are enough wells to include replicates of a media-only control condition.

***Note:*** Optimal cell densities should be determined ahead of time. Cell numbers will vary depending on the length of experiment, treatment type, and doubling time of each cell line. We recommend choosing a cell density for control (untreated) cells that remains within the linear range of cell growth, to avoid over confluency at the allotted time points (i.e., at each time point, control cells should be within 50%–80% confluency). For earlier time points (6–24 h), higher cell densities should be used (8,000–12,000 cells per well), while later time points (48–144 h), lower cell densities should be used (500–1,000 cells per well). For treated cells, cell density will depend on the treatment dose and expected kill rate. We recommend choosing a high enough density to ensure changes in cell death can be accurately monitored.

1. Day 1: Prepare cultured cells according to their growth requirements (i.e., grow the HNSCC cell line, Cal33, in Dulbecco’s Modified Eagle’s Media [DMEM] + 10% fetal bovine serum and 1% penicillin/streptomycin).
2. Working in a laminar flow hood, aspirate media from cells and wash cells with 5–10 mL of sterile 1× PBS. Add 2 mL 0.25% Trypsin (2 mL per 10 cm plate), place the plates in a 37°C incubator and wait for cells to detach (~5 min).
3. Add 5–10 mL fresh media into trypsinized cells and transfer total volume into 15 mL falcon tube. Centrifuge at 4°C, 5 min, 300 × *g*.
4. Aspirate media and resuspend in fresh media (volume will depend on confluency of the cells, i.e., 5 mL of fresh media for cells at 70% confluency). Count the total number of viable cells suspended in media using Trypan Blue and determine the number of cells to seed for each condition in a final volume of 200 μL per well. Typically, 500–1,000 cells are optimal for control cells and 8,000–12,000 cells are optimal for treated cells. However, some cell lines may require higher or lower cell numbers depending on the experimental conditions.

**CRITICAL:** Ensure enough cells are included to account for biological replicates and seeding errors. We recommend calculating an excess of 10%–20% of the required cell number and media volume.

5. Seed cells into wells of a 96-well plate using an 8-channel multichannel pipette to optimize seeding and minimize variation between wells. We recommend excluding outer wells to avoid edge effects. Include 3–4 wells of media only (blank controls) and fill unused wells with 200 μL of 1× PBS to minimize uneven evaporation. Place 96-well plates into 37°C incubator and allow cells to adhere ~2 h.
6. When cells are fully adhered, proceed with treatment. Treatment can entail either single dose IR or single dose treatment with an anti-cancer agent. When treating with IR, take care to minimize time plates are removed from 37°C incubator. Unirradiated plates should be removed from 37°C incubator for same amount of time as irradiated plates. If treating cells with a drug, remove the corresponding volume of media to ensure the drug volume does not exceed 200 μL. (Addition of small volumes [i.e., 10 μL or less] does not require removal of media).

### Measuring Cell Viability and Media Collection

**Timing: 2–3 h**

This section describes the steps used to longitudinally measure cell viability and caspase activity; and serially collect cultured cancer cell line media from the cancer cells seeded and treated above (“Seeding Cell Lines and Treatment”).

***Note:*** Prepare 8-strip PCR tubes ahead of time with labels. We recommend utilizing an appropriately sized rack or empty tips boxes to hold the tubes.

***Note:*** If using CaspaseGlo® 3/7 assay, ensure reagents are pre-mixed and aliquoted prior to use (see Promega protocol). We recommend aliquoting in 5 mL tubes and storing at −20°C until required.

***Note:*** All experiments will be performed in the original 96-well plates with seeded/cultured cancer cells. No additional 96-well plates are required for downstream assays (i.e., Presto Blue and CaspaseGlo® 3/7 assay).

7. Measure cell viability using Presto Blue protocol (Thermo Fisher). Remove and discard 20 μL of media from each well of the 96-well plate (including blank controls cells with media only) and add 20 μL of Presto Blue reagent using an 8-channel multichannel pipette into the corresponding wells of the same plate. Ensure tips are changed to avoid cross contamination. Return 96-well plates to 37°C incubator and incubate for 1 h.
8. While plates are incubating, remove aliquot of CaspaseGlo® 3/7 reagent, wrap in aluminum foil to avoid exposure to light and allow to warm to 20°C–25°C. (If this step is forgotten, place tube in 37°C water or bead bath to speed up thawing).
9. Read fluorescence on plate reader set to 37°C using the following settings: Excitation (540–570 nm) and Emission (580–610 nm).

***Note:*** Keep the lid of the plate on to maintain sterile conditions. Once finished, set the plate reader temperature to 20°C–25°C.

10. Centrifuge 96-well plate (with cultured cells, media and Presto Blue reagent) at 4°C, 5 min, 200 × *g*.
11. In a laminar flow hood, using an 8-channel multichannel pipette, collect 150 μL of media containing cfDNA from the centrifuged 96-well plate and transfer to pre-labeled 8-strip PCR tubes. Media will appear a blue-purple colour based on the Presto Blue reagent. Ensure tips are changed to avoid cross contamination.

**CRITICAL:** Avoid touching the bottom of the wells with the end of the tips to avoid dislodging of cells.

12. Once all samples are collected, immediately store tubes at −20°C until ready for downstream analysis.

***Note:*** If not measuring caspase activity, stop here.

13. Once media has been collected, using an 8-channel multichannel pipette, add 50 μL of CaspaseGlo® 3/7 reagent to each well of the 96-well plate. Ensure tips are changed to avoid cross contamination. Cover plate with aluminum foil, gently mix contents of wells using a plate shaker at 0.3 × *g* for 30 s (we used VWR Microplate shaker) and incubate at 20°C–25°C for 1 h.
14. Read luminescence on a plate reader as directed by the luminometer manufacturer (TECAN Infinite M200Pro).

**Pause Point:** Cell line media containing cfDNA can be stored at −20°C until ready to be processed. Be sure to dilute all samples from a single experiment in 1 day in order to minimize potential batch effects.

### Diluting Media

**Timing: 1–2 h**

This section describes the steps used to dilute cell line media samples with DNA suspension buffer, as detailed in our recent publication (Rostami et al. 2020).

15. Remove media samples from −20°C freezer and place on ice to thaw.
16. While samples are thawing, label 8-strip PCR tubes.
17. Add 148.5 μL of DNA suspension buffer to each tube using an 8-channel multichannel pipette.

***Note:*** Prior to aliquoting DNA suspension buffer into tubes, determine amount of DNA suspension buffer required for the total number of samples and transfer to 50 mL falcon tube. To avoid contamination, open DNA suspension buffer stock in laminar flow hood.

18. Once samples are thawed, vortex well and quick spin to remove media from the lid. Add 1.5 μL of sample to pre-labeled tubes using an 8-channel multichannel pipette. Ensure tips are changed to avoid cross contamination.
19. Store diluted samples at −20°C until ready to quantify cfDNA.

**Pause Point:** Diluted cell line media can be stored at −20°C until ready to be processed. When planning qPCR quantification of multiple time points, conditions and cell lines, try to include all time points of a single cell line and condition on one plate in order to minimize potential batch effects.

### Injection of Cell Line Suspensions into NSG Mice

**Timing: 2–3 h**

This section describes the steps for preparation and injection of human cancer cell lines into NSG mice. The following steps were used to establish mouse xenografts from our recent publication (Rostami et al., 2020).

***Note:*** Prior to cell injection, blood should be collected from mice as a pre-injection collection to establish baseline human DNA contamination levels. See “Blood Collection and Plasma Processing” for details.

20. Steps 1–3 describe cell line preparation. Aspirate media and resuspend in fresh media (volume will depend on confluency of the cells, i.e., 5 mL of fresh media for cells at 70% confluency). Count the total number of viable cells suspended in media using Trypan Blue and determine the number of cells to inject per mouse. Typically, between 150,000 and 5 × 10^6^ cells per mouse are enough to ensure tumor growth. (This will vary on the cell line and should be determined ahead of time).

**CRITICAL:** Preparation of a larger cohort of mice is suggested when possible depending on the number of cells available to ensure the sample size per treatment group is large enough and sufficient numbers of mice grow tumors.

21. Transfer the total volume of cell suspension required to a new falcon tube and centrifuged at 4°C, 5 min, 200 × *g*.
22. Cold Matrigel will be prepared at a 1:4 ratio with cold 1× PBS and mixed with the cell suspension in a 5 mL tube.

***Note:*** Matrigel matrix contains extracellular matrix components that allow for the cells to remain localized at the injection site.

**CRITICAL:** It is essential that the cell/Matrigel mixture be kept cool on ice as Matrigel forms a gel above 10°C.

23. A volume of 100 μL total is injected per mouse.
24. Aspirate the media and resuspend the cells in cold Matrigel-1X PBS solution for all mice in one tube in order to reduce variation from mouse to mouse. Be sure to prepare a few extra doses due to loss of sample in the needle hub.
25. Before filling the 0.5 mL syringe with needle attached, ensure Matrigel plus cells are mixed well by tapping the tube and/or inverting it a few times. Keep on ice.

**CRITICAL:** It is essential to thoroughly mix the cold cell/Matrigel mixture immediately before injections to ensure even distribution of cells as they will settle to bottom of tube.

26. Fill the syringe with cell/Matrigel mixture at the time the mice are ready to be injected. If the syringe is prepared too far in advance, the cells will begin to settle and become trapped inside the hub.
27. Hold the mouse in an Intraperitoneal (IP) hold and subcutaneously inject 100 μL into the right or left flank of each mouse.

***Note:*** If uncomfortable with injecting the mice while awake, place mice under isoflurane prior to injection.

***Note:*** Each mouse should be ear notched either prior to cell injection or immediately afterwards to ensure identification.

### Irradiation of Cell Line Xenograft Tumors

**Timing: 3–5 h**

This section describes the steps that were used to treat HNSCC xenograft mice with irradiation in our recent publication (Rostami et al., 2020). This protocol can be used when treating with other drugs of interest, using the correct vehicle, dose, and delivery route that is appropriate.

28. Monitor the tumor growth of each mouse by palpation and caliper measurements. At each tumor measurement, blood can be collected to monitor human cfDNA levels during tumor development. See “Blood Collection and Plasma Processing” for details.
29. Using calipers measure and record the measurements for each tumor (length (L), width (W) and height (H), mm).
  a) Volume = 4/3 × PI × (L/2 × W/2 × H/2) (where PI = 3.14159)
30. Treatment will begin when the volumes of the tumors reach approximately 80–200 mm^3^.

**CRITICAL:** It is important that all tumors are approximately of similar volume.

31. Randomize the mice into treatment groups (5–10 mice per group, irradiated vs untreated).
32. Prior to irradiation, collect an aliquot of blood (See Blood Collection and Plasma Processing” for details) which will serve as the Pre-irradiation blood collection (0 h or day 0).

**CRITICAL:** Blood should be collected right before treatment, if possible 1–2 h before. Since all longitudinal blood draws will be compared back to Pre-irradiation, this timing will ensure an accurate pre-treatment human cfDNA baseline level.

33. Tumors are irradiated according to the guidelines of the irradiator. We used an IGRT-XRAD 225Cs irradiator that delivered a dose of 20 Gy to the tumor (half the dose from the top and half the dose from the bottom of the tumor). The timing of IR was dependent on the collimator used and depth of tumor determined using integrated software and cone-beam computed tomography volume scans for targeting of the desired irradiation. Irradiations were performed at 225 kVp (peak kilovoltage) and 13 mA (milliampere).

***Note:*** Any small animal irradiation system can be used, but planning should be taken to minimize the time mice are removed from the facility to avoid over manipulation.

34. Once treated, mice are monitored according to the approved blood collection schedule.

***Note:*** Our animal use protocol was approved for serial blood collections at day 0, 2, 4 and 6 post-treatment (0 h, 48 h, 96 h, 144 h).

### Blood Collection and Plasma Processing

**Timing: 1–3 h**

This section describes the steps that were used to collect and process blood from xenograft mice in our recent publication (Rostami et al. 2020).

***Note:*** When handling the blood samples and processing the plasma, a surgical mask should be worn at all times in order to minimize human DNA contamination.

***Note:*** Blood collection described in this protocol is performed using a submandibular blood draw technique. This technique has been optimized in our lab and is strongly amenable to serial collection of small volumes of blood, does not require restraint devices or analgesics, and is a quick and relatively painless procedure. Other survival blood collection procedures can also be used (i.e., tail vein, saphenous vein); however, performance for consistent and reliable serial sampling may be affected.

35. At each time point, tumors are measured, and blood is collected from the submandibular vein (Golde et al. 2005).
36. Microvette CB 300 tubes coated with EDTA should be pre-labeled with corresponding mouse IDs.
37. Within a laminar flow hood, a sterile 5 mm lancet should be placed on a paper towel and tubes should be opened and ready to use. Gauze pads should be placed nearby for easy access.
38. 1 mL syringes with attached needle should be prepared with 150 μL of saline per mouse.

***Note:*** The submandibular vein can be used for serial blood collection of small samples by alternating cheek sides, and/or by gently removing the clot/scab.

***Note:*** This method can be performed without anesthesia but does require training to perfect the technique.

39. Properly restrain the animal with one hand using the scruffing technique, which will apply adequate pressure to the maxillary vein.

***Note:*** It is important that side-to-side movement of the head be minimized to ensure accurate and safe venipuncture with the lancet.

**Figure 1:**
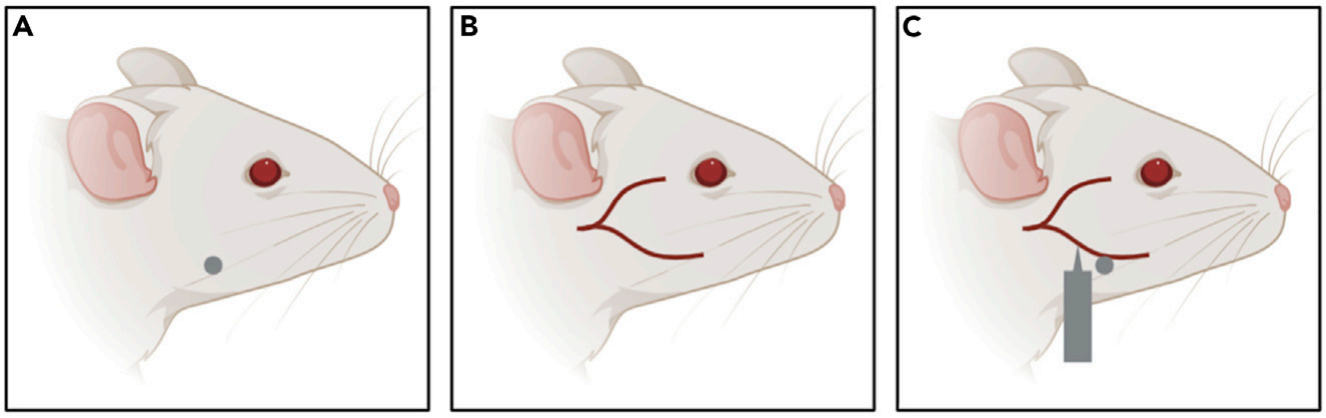
Location of Submandibular Blood Draw (A) Visualization of hairless dimple/hair whorl. (B) Location of facial veins and submandibular vein. (C) Position of lancet to puncture submandibular vein.

***Note:*** Visualize landmarks. Mice have a hair whorl or hairless dimple (Figure 1A), along the curvature of the mandible; the vein is just below this mark in the groove past the jawbone (Figure 1B).

40. Using the point of the lancet, apply firm pressure at the point, caudal to the eye and ventral to the ear, where the submandibular vein is located, then release until blood flows (Figure 1C).

**CRITICAL:** Hold lancet perpendicular to the bleed site to avoid puncturing the ear canal.

41. Quickly place the blood collection tube below the puncture site until desired volume of blood is collected. (2–3 drops are sufficient, 50–100 μL). Place tube on ice until ready to process.

**CRITICAL:** Blood should be processed within 1 h of collection

42. Apply gentle pressure with gauze until bleeding has stopped.
43. Inject 150 μL of saline IP to ensure hydration of mouse for subsequent blood draws.
44. Blood samples are centrifuged at 2,500 × *g* for 10 min at 4°C.

***Note:*** 1.5 mL microtubes should be pre-labeled prior to blood processing. We recommend using a label maker to ensure accurate identification. Two microtubes per sample should be labeled, one for plasma and one for the blood pellet.

45. The separated plasma portion should be transferred to a clean 1.5 mL microtube labeled blood pellet. The remaining blood portion should be kept on ice.
46. The new tubes with collected plasma are centrifuged at 16,100 × *g* for 10 min at 4°C to remove additional debris.
47. The entire volume of plasma should be recorded and transferred to the new microtube labeled plasma. The volume can be recorded on the side of the tube and in a lab book to ensure accuracy. The remaining blood pellet can be transferred to the corresponding microtube tubes labeled blood pellet, if desired. All tubes are stored at −80°C.

**CRITICAL:** All tubes should be kept on ice during processing. A mask should be worn when handling samples to avoid contamination from human DNA. It is important to record the plasma volume for downstream calculations.

**Pause Point:** Plasma can be stored at −80°C until ready to be purified. When purifying multiple samples, ensure appropriate planning to minimize batch effects between samples. We recommend purifying all samples from one treatment condition together.

### Cell-free DNA Purification

**Timing: 3–4 h**

This section describes the steps to purify small volumes of plasma collected from xenograft mice in our recent publication (Rostami et al. 2020).

***Note:*** This protocol is a modified version of the QIAamp 96 DNA Blood Kit (Qiagen) and uses reagents from the Qiagen Nucleic Acid Purification Kit (ACL and ACB). Prior to beginning this protocol, all reagents should be accounted for to ensure a smooth workflow.

***Note:*** This protocol is used for samples that are 200 μL or less in volume.

***Note:*** Although this kit can process up to 96 samples, we do not recommend using the entire block to avoid samples sitting for too long. The process becomes faster as you perform the protocol more often, we recommend optimizing the number of samples based on comfort level and expertise. Our maximum number of samples has been 70 samples. We recommend starting with between 20–40 samples.

***Note:*** A solution of 1× Phosphate Buffer Solution + 0.5 mM EDTA should be prepared to top up each plasma sample to a total volume of 200 μL. The volume of this solution will vary depending on the number of plasma samples and recorded volumes (For example, if each plasma sample is 75 μL and requires a top up of 125 μL of 1× PBS-0.5 mM EDTA, then 24.75 mL 1× PBS, 25 μL 0.5 M EDTA for total of 25 mL would be sufficient volume for ~200 samples).

***Note:*** We recommend printing out a sheet with sample IDs, plasma volumes and required volumes to top up to 200 μL. We also recommend printing out the layout of samples in a 96-well format to ensure accuracy when loading samples into the collection microtubes and QIAamp 96 plate.

***Note:*** When purifying the plasma samples a surgical mask should be worn at all times to minimize human DNA contamination.

48. Set oven to 60°C.
49. Remove plasma samples from −80°C and thaw on ice.
50. While samples are thawing, add 20 μL of proteinase K to the bottom of each collection microtubes (blue rack).
51. Once samples are thawed, top plasma samples to 200 μL with 1× PBS +0.5 mM EDTA solution if required. Samples can be placed in an microtube rack at 20°C–25°C.
52. Add the entire volume of plasma samples (200 μL) to each corresponding collection microtube.

***Note:*** We recommend labeling the tubes 1–8, 9–16 etc. to ensure the order of the samples is maintained.

**CRITICAL:** When pipetting samples into the collection microtubes, ensure the samples are transferred near the bottom of the tube to avoid possible loss of sample.

53. Once all the plasma samples have been added, carefully pipette 200 μL of buffer ACL (without RNAse carrier) using an 8-channel multichannel pipette.

***Note:*** Pipette slowly as the solution is quite viscous.

54. Cap the tubes carefully and apply plastic cling wrap to each 8-strip of collection microtubes to avoid spilling.
55. Vortex each strip for 15 s, pulsing.

***Note:*** Keep your hand on the top of the tubes to ensure the caps do not come off.

56. Remove the plastic cling wrap carefully and place the collection microtubes back in the rack. Balance the rack and centrifuge at the highest speed (our centrifuge was set to 3,468 × *g*) for 1 min to collect any solution from the caps.

**CRITICAL:** Due to the high spin force, balancing within the centrifuge is extremely important. We ensure accurate balancing by weighing the tube rack on a scale and adjusting for balance with water.

57. Following centrifugation, wrap each strip of collection microtubes in plastic cling wrap, and place in the 60°C oven to incubate for 30 min.

***Note:*** An incubator or oven is recommended for this step based on the manufacturers protocol. The use of a water bath has not been tested in our lab; thus, caution should be taken if performing this step with an alternative heat source.

58. Repeat step 56. Turn oven to 56°C.
59. Carefully uncap the tubes, placing the caps upwards to avoid contamination. Carefully pipette 200 uL of ACB buffer using an 8-channel multichannel pipette.
60. Repeat steps 54–55. Immediately after vortexing, place tubes on ice for 5 min.
61. Repeat step 56, adjusting balance accordingly.

**CRITICAL:** Due to the high spin force, balancing within the centrifuge is extremely important. We ensure accurate balancing by weighing the tube rack on a scale and adjusting for balance with water.

62. Set-up the 96-well Vacuum Manifold (we use the NucleoVac 96 Vacuum Manifold with Square-block spacer). Insert the appropriate spacers in the manifold base. Place an S-block on top to collect waste. Place the manifold lid on top of the manifold base. Place the QIAamp 96 plate on top of the manifold lid.

***Note:*** If using a different manifold, ensure the QIAamp 96 plate is correctly positioned over the S-block.

63. If there are unused wells on the QIAamp 96 plate, cover them with a plastic seal to avoid contamination and enable future use.
64. Carefully apply the entire mixture (620 μL per collection microtube) to the QIAamp 96 plate. Repeat until all samples have been transferred to the QIAamp 96 plate.

**CRITICAL:** Carefully remove the caps to avoid cross contamination. We recommend using a P1000 to transfer the samples to the plate and then switching to a P200 to transfer any residual sample. Begin drawing up the samples when the pipette tips contact the liquid to avoid overflow by lowering the tips too far into the tube.

65. Seal the QIAamp 96 plate with an AirPore Tape sheet. Turn on the vacuum and allow the sample to flow through the column to allow binding.

***Note:*** Pressure may be required to allow a seal on the vacuum to form. Press down gently on the QIAamp 96 plate allow suction.

66. Carefully remove the QIAamp 96 plate from the manifold and place between two tip boxes to avoid contamination of samples.

**CRITICAL:** This step requires careful attention to avoid contamination. Ensure there is enough space on your workbench and prepare two tip boxes to balance the QIAamp 96 plate without touching the bottom of the plate.

67. Carefully empty the flow-through waste in the S-block using an aspirator.

**CRITICAL:** This step requires careful attention to avoid contamination.

68. Return the QIAamp 96 plate to the manifold. Add 500 μL Buffer AW1 using an 8-channel multichannel pipette to each well. We recommend adding 250 μL in two additions using a 300 μL multichannel pipette.
69. Repeat step 65.
70. Add 500 μL Buffer AW2 using an 8-well multichannel pipette to each well. We recommend adding 250 μL in two additions using a 300 μL multichannel pipette.
71. Repeat step 65.
72. Repeat steps 66–67. Remove the S-block from the manifold and carefully return the QIAamp 96 plate directly on top of the S-block in the correct position.
73. Weigh the QIAamp 96 plate/S-block and adjust the balance. Centrifuge at the highest speed (our centrifuge was set to 3,468 × *g*) for 10 min.

**CRITICAL:** Due to the high spin force, balancing within the centrifuge is extremely important. We ensure accurate balancing by weighing the tube rack on a scale and adjusting for balance with water.

74. Remove the QIAamp 96 plate from the S-block and place between two tip boxes. Incubate in the oven at 56°C for 10 min.

**CRITICAL:** This step requires careful attention to avoid contamination. Ensure there is enough space in the oven and prepare two tip boxes to balance the QIAamp 96 plate without touching the bottom of the plate.

75. Place the QIAamp 96 plate directly on top a rack of elution microtubes. Add 100 μL of EB buffer at 20°C–25°C using an 8-channel multichannel pipette to each well of the QIAamp 96 plate. Seal the QIAamp 96 plate with a new AirPore Tape sheet and incubate for 10 min at 20°C–25°C.

***Note:*** EB buffer should be carefully added to the center of the wells to ensure maximum elution.

76. Weigh the QIAamp 96 plate/rack of elution microtubes and adjust the balance. Centrifuge at the highest speed (our centrifuge was set to 3,468 × *g*) for 12 min.

**CRITICAL:** Due to the high spin force, balancing within the centrifuge is extremely important. We ensure accurate balancing by weighing the tube rack on a scale and adjusting for balance with water.

77. Transfer the entire volume from each elution microtube into new labeled tubes. Due to some loss of sample, elution volumes will range between ~80–90 μL. You can record the exact volume for each sample if time permits. Based on our results, we estimate an elution volume of 85 μL for downstream calculations. Store purified cfDNA samples at −20°C.

***Note:*** Tubes should be pre-labeled ahead of time. Ensure samples are transferred to the appropriate tubes. Having a printed layout of the position of each sample on the QIAamp 96 plate ensures accuracy. We recommend using 8-strip PCR tubes.

### Quantifying Cell-free DNA by qPCR

**Timing: 3–4 h**

This section describes the steps for quantification of human cfDNA from diluted cell line media and purified plasma. Human cfDNA is quantified using qPCR targeting human LINE-1 (hLINE-1) (Rago et al. 2007). hLINE-1 is a retrotransposon family member with ~100,000 elements interspersed throughout the human genome. Due to the presence of many repeats, hLINE-1 enables ultrasensitive quantification of human DNA in small volumes of mouse plasma and diluted cell line media.

***Note:*** The qPCR set-up should be performed in a qPCR hood when available to avoid contamination of human DNA.

***Note:*** This qPCR protocol was designed for 10 μL reaction in 384-well format using SYBR Green super mix (Bio-Rad) and a CFX384 Touch Real-Time PCR Detection system (Bio-Rad). The exact procedure differs depending on the reagents and machine used, but the general design principles are the same for all qPCRs.

***Note:*** A human genomic standard dilution series should be made ahead of time. When comparing multiple qPCR plates, the same material should be used for all standard curves to allow accurate comparison with each other. We suggest making a stock of each dilution and aliquoting into 8-strip PCR tubes, to be stored at −20°C. Ensure each aliquot has enough material for three technical replicates of 4 μL each + 20%–30% of total volume. We use a 5-step 10-fold dilution series between 0.0125 pg/μL and 125 pg/μL. Ensure standards are made in the appropriate solutions based on the samples (i.e., for diluted media samples, genomic standards should be prepared in DNA suspension buffer and for the purified plasma cfDNA samples, genomic standards should be prepared in EB buffer).

***Note:*** Two nested hLINE-1 primer sets can be used to quantify cfDNA. The short hLINE-1 primer set will amplify fragments 82 base pairs or larger. The long hLINE-1 primer set will amplify fragments 224 base pairs or larger. An integrity index can be calculated as the ratio of long hLINE-1/short hLINE-1. Both primer sets are specific to hLINE-1 and do not have homology to mouse, bovine, or rabbit genomic DNA.

78. Thaw (a) diluted cell line media samples or (b) purified plasma cfDNA samples on ice along with forward and reverse primer stocks and pre-made human genomic standard dilution series.
79. Make a working stock of the hLINE-1 primer pair combination by adding the forward and reverse primers to a final concentration of 2.5 μM (For example, add 180 μL MQ, 5 μL of 100 μM forward primer and 5 μL of 100 μM reverse primer for a 200 μL stock). Mix well by vortexing.
80. Make a master mix of 5:1 SYBR Green super mix to 2.5 μM hLINE-1 primer working stock for the number of desired reactions + 10%. (For example, add 550 μL SYBR Green, 110 μL 2.5 μM hLINE-1 primer working stock for 100 reactions).
81. Vortex the thawed diluted samples and SYBR Green-hLINE-1 primer master mix to ensure they are mixed well.

***Note:*** Do not vortex genomic standards.

82. Working in the qPCR hood, place a Kim wipe over top an ice box and place a 384-well plate on top. Prepare the qPCR plate by marking which wells will be used.

***Note:*** To keep track of which wells are to be filled and which are not, we recommend printing out the plate layout and marking after each sample addition.

83. Add 6 μL of the SYBR Green-hLINE-1 primer master mix to wells of the 384-well plate. It is recommended to use a repeat dispenser to increase speed and accuracy (we used Repeater® M4).
84. Add 4 μL of each purified plasma or diluted cell line media sample to the wells of the 384-well plate. It is recommended to use an electronic pipette to increase speed and accuracy (we used Viaflo Electronic Pipette).

***Note:*** We recommend using three to four technical replicates per sample.

***Note:*** Avoid sample contamination by hovering the tip over the wells of the 384-well plate and carefully dispensing the sample onto the side of the well.

85. Create the genomic standard curve by adding 4 μL of the 10-fold genomic standard dilution series to the wells of the 384-well plate. Ensure standards are mixed well by flicking tubes gently. Also fill three wells with DNA suspension buffer as a no template control (NTC).

***Note:*** We recommend using three to four technical replicates per sample.

86. When all the samples are loaded, seal the plate properly with a seal compatible with qPCR.

***Note:*** Check the seal for damage or dirty spots. The qPCR machine will measure the fluorescence through the seal, anything on the seal that changes the path of the light can affect the measured values.

***Note:*** To ensure all material is at the bottom of the well, centrifuge briefly for 1 min at 200 × *g*.

87. Run the qPCR using an appropriate program. An example of our protocol is shown below.

**Table:** 

Step	Temperature	Time
1	98°C	3:00
2	98°C	0:10
3	60°C	0:30 + measure fluorescence
4	Go to step 2, 39 more times	
5	65°C	0.05
6	95°C	0.5

***Note:*** Ensure the standard curve is set to the corresponding amounts (pg) using in the dilution series (i.e., for 4 μL per well, the standard curve should be set to 0.05–500 pg).

88. When the qPCR run is complete, ensure the standard curve has a R^2^ value >0.9. Next ensure the sample Cq values fall before the NTC (i.e., are above background). After exporting the data to the format of choice (for example excel file), calculate the starting quantities (SQs) of all reactions using the corresponding standard curve (the Bio-Rad CFX Maestro software will do this automatically).
89. Calculate the SQ of each well by dividing by the sample input volume (4 μL) and multiplying the SQs by the dilution factor (i.e., 100 X) if any.
90. Average the SQ of each sample by averaging the replicates.

***Note:*** Sometimes a reaction of a single replicate can fail. If there is a clear single outlier, this outlier can be removed to get a more accurate estimate of the SQ.

91. Normalize and calculate the final cfDNA concentration of each sample.
  a) For diluted media samples, normalize the cfDNA concentrations by the initial seeding density. If the same seeding density was used for all conditions, this step can be omitted. Average the normalized cfDNA concentration of the biological replicates of the control samples. To calculate the fold change in cfDNA release divide the normalized cfDNA concentration from treated samples to the corresponding averaged control samples.
  b) For plasma cfDNA samples, the absolute cfDNA concentrations (ng/mL) can be calculated using the following equation (Lo et al., 2000): C = Q × V_DNA_/V_qPCR_ × 1/V_ext_, where C = target cfDNA concentration in plasma (ng/mL), Q = starting quantity (pg/uL) determined by qPCR , V_DNA_ = total volume of DNA obtained after extraction (approximately 85 μL), V_qPCR_ = volume of DNA solution used for qPCR (4 μL), and V_ext_ = volume of plasma extracted. cfDNA concentrations are normalized to tumor volume (mm^3^) at each timepoint to derive “cfDNA density,” which is compared to Pre-irradiation (0 h) at all time points.

## Expected Outcomes

**Figure 2:**
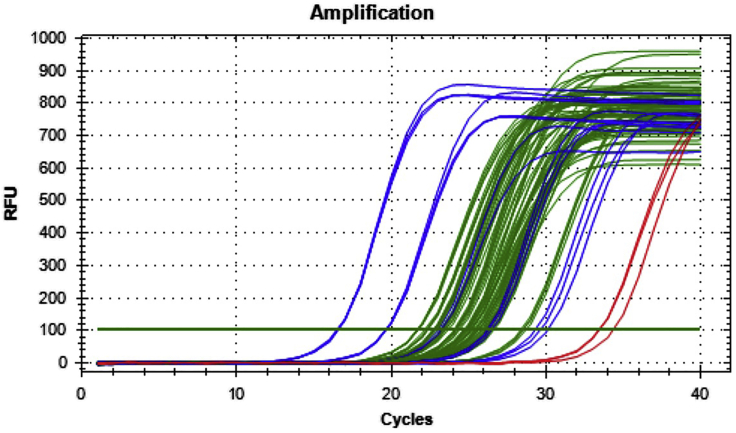
Example of Raw qPCR Data from a Successful Run Blue lines are genomic standards, red lines are the NTC, and green lines are the samples.

A successful qPCR for human cfDNA should show Cq values above the background non-template control (NTC) and within the genomic standards. cfDNA yields (pg) for diluted culture media and xenograft plasma samples will vary depending on the cell line, conditions, and time points. An example of a typical qPCR run is shown in Figure 2. Pre-treatment absolute cfDNA concentrations (after accounting for original plasma volume and elution volume) across five different xenograft models ranged between 0.02–1.3 ng/mL from tumors between 80–200 mm^3^ and plasma volumes between 12–106 μL.

## Quantification and Statistical Analysis

**Figure 3:**
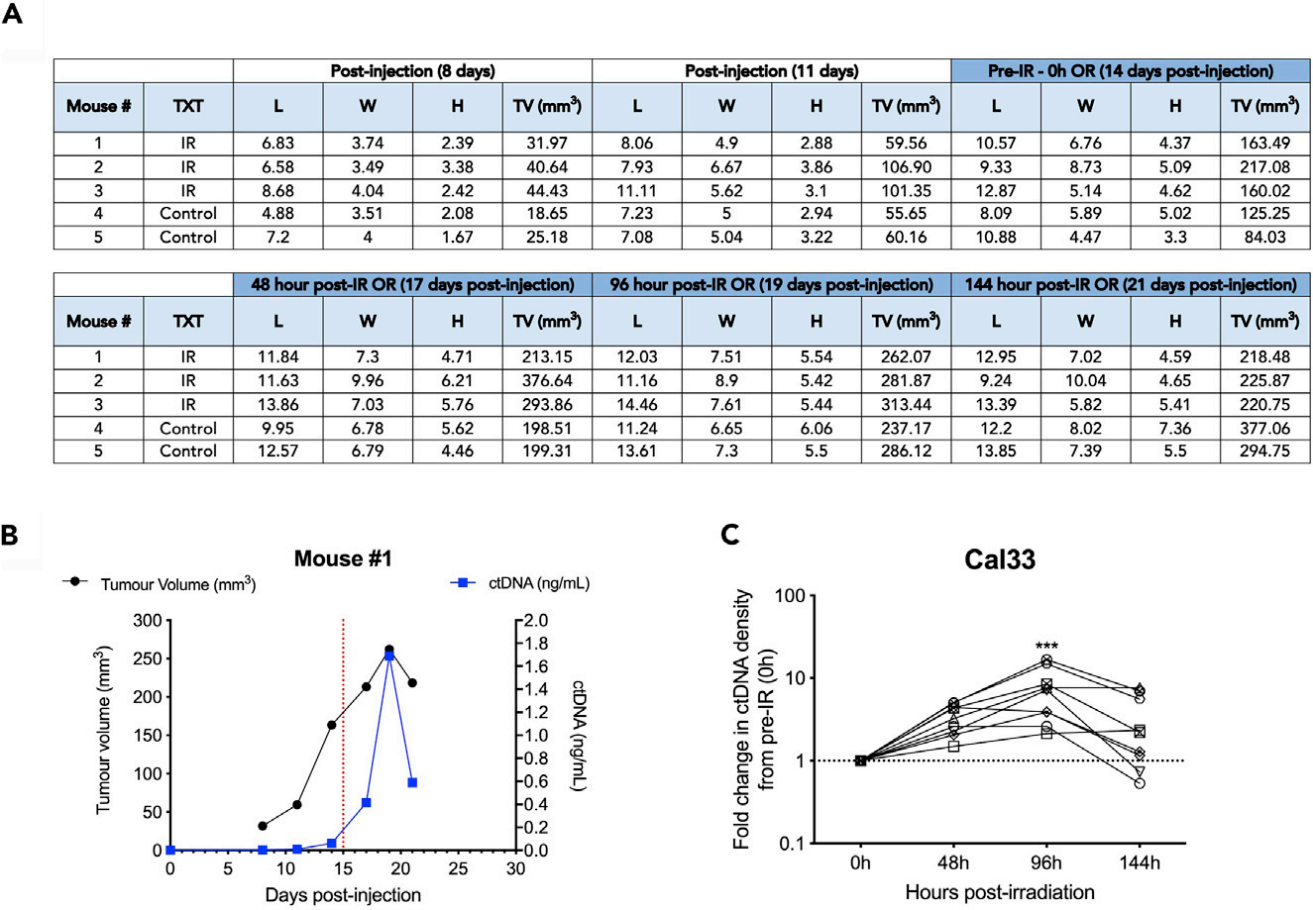
Record of Tumor Measurements and Data Visualization Post-cfDNA Quantification (A) An example of a spreadsheet used to record the tumor sizes during treatment and blood collections. (B) A summarized graph showing the tumor volumes and absolute cfDNA concentrations (ng/mL) over time. Red dotted line denotes the initiation of treatment. (C) An example of the final analyzed “cfDNA density” metric compared to 0 h. Statistical analysis used is a one-way ANOVA with Dunnett’s multiple comparison. IR, ionizing radiation; L, length; W, width; H, height; TV, tumor volume; TXT, treatment. From Rostami et al. (2020).

1. It is useful to create a file that can be used to record tumor measurements and results during the tumor growth and treatment phase (Figure 3A).

2. The tumor volumes and cfDNA concentration can be plotted for each mouse to evaluate the kinetics of cfDNA release and tumor growth over time (Figure 3B).
3. To evaluate the kinetics of cfDNA release within each treatment arm, the cfDNA density metric can be calculated and the fold change in cfDNA density from 0 h (Pre-irradiation) can be plotted for each mouse (Figure 3C).
4. A repeated measures 1-way ANOVA (with multiple comparisons correction) can be used for statistical analysis between serial timepoints compared to 0 h.

## Limitations

One limitation of the serial assessment of cfDNA in preclinical models is that certain cell types may not shed detectable levels of cfDNA. Although this biological phenomenon is an important characteristic and finding, some cell lines of interest may not successfully be utilized for cfDNA release evaluation. We see good concordance between cfDNA detection *in vitro* and *in vivo*, thus we recommend evaluating cfDNA release from cell lines of interest in culture before moving to xenograft models. Another limitation is the confounding effects of tumor characteristics such as cystic or necrotic tumors on cfDNA release. We initiated treatments on tumors between 80–200 mm^3^ to minimize the possibility of necrotic tumor cores and development of cystic tumors. However, some xenograft models may not be suitable for studies depending on their growth characteristics. We recommend collecting endpoint tumors for immunohistochemical staining to identify possible mechanisms that might be contributing to any cfDNA release kinetics you observe. As previously stated, the use of immunocompromised mice limits the ability to assess the function of the immune system in modulating cfDNA release, degradation, and clearance. This limitation should be considered when utilizing these models for cfDNA analysis. Additionally, as previously indicated, caution should be taken when performing this protocol with media types and DNA polymerases not tested in our lab. Lastly, our quantification of cfDNA relies on the high sensitivity of hLINE-1 from cell line xenograft tumors. This method of quantification is not directly translatable to syngeneic tumor models, where hLINE-1 cannot differentiate between tumor-derived cfDNA from normal cfDNA.

## Troubleshooting

### Problem 1

Results from qPCR quantification show large variations between biological replicates, with some replicates with large cfDNA yields compared to other samples (steps 88–90).

### Potential Solution

When collecting the media at each time point, it is important to slowly collect media without touching the bottom of the wells (step 11). Sometimes, dislodged cells can be transferred with the media and burst open, contributing to increased levels of cfDNA. Try to hover the pipette tips near the side of the wells, over the bottom of the plate. If there are samples with obvious variations from other biological replicates, it is also okay to omit these samples from the average. This is why we recommend using 3–4 biological replicates.

### Problem 2

High background human DNA in NTC (step 88).

### Potential Solution

Due to the sensitivity of the qPCR assay, minute amounts of human DNA will be detected. The NTC is included to account for the background levels of human DNA. If the DNA suspension buffer or EB buffer is opened outside a laminar flow hood or used by multiple lab members, the buffers can become contaminated with higher levels of human DNA. We suggest aliquoting the buffers to minimize over handling and maintain sterile conditions when opening the stocks.

### Problem 3

During injections of cell line cancer cells, cells can leak out (step 27).

### Potential Solution

Loss of material can happen for a variety of reasons; however, this is most likely to occur due to the speed and force of injection. To avoid loss of cells, inject the cells slowly into the pocket of skin. Be sure to have enough loose skin lifted on the flank to create the pocket. Inject the cells and hold the needle for 3–5 s, remove slowly by turning the needle gently while pulling out of the injection site. We recommend anesthetizing the mouse if issues arise with proper restraint of the mouse during injection.

### Problem 4

No detectable cfDNA following qPCR quantification of purified plasma samples (steps 88–90).

### Potential Solution

Some tumors do not release cfDNA or release very low levels (see our recent publication (Rostami et al. 2020)). To confirm, plasma collected from an endpoint cardiac puncture can be purified and human-derived cfDNA can be quantified. Using larger volumes of plasma might increase the sensitivity of detection of low shedding tumors and answer this question. However, it is possible that despite increased plasma volumes human-derived cfDNA remains undetectable for certain tumors.

### Problem 5

Plasma volumes are too large (>200 μL) for purification (step 51).

### Potential Solution

In the event that more than 200 μL of plasma is collected from a mouse, the sample should be divided into equal volumes and treated as individual samples throughout the protocol (i.e., added to separate collection tubes, columns of the QIAamp 96 plate etc.). The cfDNA yield quantified by qPCR for the divided samples can be added together to determine the total amount (pg).

## Resource Availability

### Lead Contact

Further information and requests for resources and reagents should be directed to and will be fulfilled by the Lead Contact, Scott Bratman (scott.bratman@rmp.uhn.ca).

### Materials Availability

This study did not generate new unique reagents.

### Data and Code Availability

This study did not generate any unique datasets or code. The authors declare that the data supporting the findings of this study are available within the paper.
